# Therapeutically-induced stable disease in oncology early clinical trials

**DOI:** 10.1371/journal.pone.0233882

**Published:** 2020-05-29

**Authors:** Francois Mercier, Georgina Meneses-Lorente, Paul Grimsey, Alex Phipps, Francesca Michielin

**Affiliations:** 1 Department of Biostatistics, Roche Innovation Center Basel, Basel, Switzerland; 2 Department of Clinical Pharmacology, Roche Innovation Center Welwyn, Welwyn Garden City, United Kingdom; Tata Memorial Centre, INDIA

## Abstract

**Rationale:**

The RECIST guideline defines four categories of response to treatment for cancer patients according to post-baseline changes in tumor burden, hence ignoring disease history. However, if left untreated, tumors grow exponentially, implying that pretreatment changes in tumor size are key to thoroughly assess efficacy. We present a model-based approach to estimate the rates of changes in tumor mass, before and after treatment onset.

**Methods:**

Sixty-eight patients were eligible for the analysis of tumor size data from a Phase 1 study evaluating the effect of emactuzumab. In addition to tumor size measured at baseline and every six weeks during treatment, a pre-baseline measurement was gathered for each patient. A longitudinal regression model was used to estimate the rates of tumor size change before and after treatment onset.

**Results:**

The median pre-treatment tumor growth exponential rate was equal to 0.022 month^-1^, corresponding to a tumor size doubling time of 4 months, and the on-treatment median tumor shrinkage exponential rate was equal to 0.001 month^-1^. Among sixteen patients categorized as stable disease per RECIST, only five had similar slopes before and after treatment while nine actually improved. One patient in particular had a therapeutically induced stabilization of the disease.

**Conclusion:**

Our analysis emphasizes the importance of collecting pre-baseline scans to distinguish therapeutically induced stable disease from cases where the tumor growth is not perturbed by treatment.

## Introduction

Adopted by the pharmaceutical industry for the past 20 years, the Response Evaluation Criteria In Solid Tumors (RECIST) guideline [1,2] has established itself as a standard way to evaluate response to therapeutic treatment of solid tumors in clinical trials. In this guideline, the sum of (target) lesion diameters (SLD) is taken as a measure of tumor burden. SLD is measured at the baseline visit scheduled a few days to a few weeks before treatment onset and followed regularly thereafter. Typically, on-treatment computed tomography (CT) or magnetic resonance imaging (MRI) scans are taken every 6 to 12 weeks until disease progression or end-of-study. Time profiles of SLD values are used to evaluate the changes in tumor burden due to treatment. The RECIST guideline defines four categories to rank the antitumor response at each visit: complete or partial response (CR or PR), and stable or progressive disease (SD or PD). Responding patients are those who achieve at least a 30% reduction of SLD during the treatment period compared to baseline. Stable disease covers a wide range of SLD changes that encompass a 29% decrease in SLD all the way to a 20% increase from nadir (which is not necessarily the baseline). The imprecision in the term “stable disease” (SD) has resulted in some clinicians having little confidence in interpreting it, or even considering it as indicating a failure of treatment. However, it has been reported that the growth of a tumor mass in untreated patients follows approximately an exponential function [3]. Thus, an on-treatment stable disease could already be indicative of a successful therapeutic modality.

In recent publications, Ferté and colleagues [4,5] have advocated in favor of adding a pre-baseline (CT or MRI) scan to the set of scans collected during oncology clinical trials, which, to date, had only routinely included baseline and on-treatment scans. For each patient, a reference or pre-treatment exponential rate of change in tumor size was derived from the observed pre-baseline and baseline scans, and similarly, an experimental or on-treatment exponential rate of change in tumor size was derived from the baseline and first on-treatment scan. Negative values of exponential rate would be interpreted as tumor shrinkage, and positive values, as tumor growth. More importantly, negative values of difference (or ratio smaller than 1) between on-treatment and pre-treatment rates would be interpreted as a declination of the tumor burden upon treatment onset, and vice-versa, positive values of difference (or ratio greater 1) would correspond to an inclination of the tumor burden.

The approach including the pre-baseline scan, therefore, gives more information about the effectiveness of the investigative medicine given individual differences in tumor characteristics. It is particularly interesting in the case of patients classified as SD according to RECIST. Indeed, the difference (or ratio) between the pre- and on-treatment exponential rates could enable to identify *therapeutically induced stable disease* (when the growth rate of an aggressive tumor would be slowed down upon treatment initiation) distinguishing it from cases where the tumor growth is not perturbed by treatment.

Ferté and colleagues have offered an original and easy-to-implement solution to assess the difference in tumor size dynamics before and after treatment onset. Their approach improves upon the pragmatic assumptions of tumor homeostasis inherent to the RECIST guideline, however it has some limitations. Firstly, the date of ‘baseline’ scan was assumed to coincide with the date of treatment onset, which rarely reflects clinical practice. The baseline scan is typically taken a few days to a few weeks before treatment onset, a period during which the tumor could have grown. Hence, the tumor size measured at the baseline visit by these authors may be different from the size at the time of treatment onset. Secondly, it was assumed that the on-treatment rate derived from the first tumor assessment only was an adequate estimator of a long term trend; hence, ignoring all possible information coming thereafter.

In this article, we present a model-based approach to estimate the rates of changes in tumor mass, before and after treatment onset. Meanwhile, we address the challenges identified in the analysis reported by Ferté and colleagues. We apply this model-based approach to assess the efficacy of emactuzumab [6] (a.k.a. RG7155) in solid and soft tissue tumor patients. We present the results and discuss in detail the new perspective brought by this approach on the potential benefits derived by the patients from the investigated treatment, and critically review the case of patients classified as SD according to RECIST. Based on this work, we draw some conclusions on the advantages and limitations of having a pre-baseline scan in early oncology clinical trials.

## Materials and methods

### Trial and patient selection

The entry-into-human study NCT01494688 was designed to assess the efficacy, safety and pharmacokinetics of the CSF1R inhibitor emactuzumab as monotherapy or in combination with paclitaxel. The ethical approval was waived because the full details of the design and main results of the study were reported in previous manuscripts [7,8]. The monotherapy (on which we focus in this report) was first evaluated in a dose escalation, and then in an expansion cohort, where patients received what was considered to be the optimal biological dose. In the expansion cohort, patients with Pigmented Villonodular Synovitis (PVNS), soft tissue sarcoma or malignant mesothelioma, locally advanced and/or metastatic ovarian (including fallopian tube), endometrial or breast cancer and pancreatic cancer were treated. Patients had to have a histologically confirmed diagnosis, ECOG performance status of 0 or 1, measurable disease according to RECIST1.1 [2] as assessed by the investigator and they had to have no prior chemotherapy, radiotherapy or any investigational treatment within 28 days of first receipt of emactuzumab. Our work is a retrospective analysis of the data collected in Part II of the expansion cohort (as defined in S1 Fig of [8]).

Radiological assessments for all patients were scheduled at baseline and every six weeks thereafter, until end-of-study or disease progression. The scans were evaluated by both the investigator and a blinded central independent review (BICR); in our analysis, we use the latter. The best overall response (BOR, as defined per RECIST1.1 guideline) was determined at the end of study for each patient respectively. In addition, the last pre-baseline scan was collected to assess tumor growth rate prior to start of investigational treatment using the same BICR facility.

### Statistical analysis

For the patients who had at least two MRI scan evaluations before the start of the treatment (a pre-baseline scan and a baseline scan) and at least one tumor assessment (TA) after treatment onset, the tumor dynamics could be modelled through longitudinal segmented line regression model (Eqs (1) and (2)) [9].

$$y_{ij}=y_{0i}∙exp(r_{gi}t_{ij}∙I(t_{ij}\leq 0)+r_{si}t_{ij}∙I(t_{ij}>0))∙exp(\varepsilon_{ij})$$ (1)

which can be re-expressed in the logarithmic domain as:

$$y_{ij}^{\prime }=ln(y_{ij})=ln(y_{0i})+r_{gi}t_{ij}∙I(t_{ij}\leq 0)+r_{si}t_{ij}∙I(t_{ij}>0)+\varepsilon_{ij}$$ (2)

with *y*_*ij*_, the patient SLD (in mm) observed at the *j*^th^ occasion in patient *i*, *t*_*ij*_, the time in month, *r*_*gi*_, the slope before time 0, *r*_*si*_, the slope after time 0, and *y*_0*i*_, being the intercept, i.e. the estimated SLD value for patient *i* at time 0. *I*(∙) is an indicator function equal to 1 when the statement is true. The residual error terms *ε*_*ij*_ were assumed to be normally distributed with mean 0 and pooled standard deviation *σ_res_*. The time 0 was set by the treatment start, and served as fixed breakpoint in the segmented regression model; hence the difference in slopes expressed the treatment effect. Details on the software and algorithm used to fit the segmented line regression model (Eq (2)) are provided in the S1 Text. The statistical analysis was performed in R (version 3.1.0), using the lmList function of the “nlme” R package [10].

**Fig 1:**
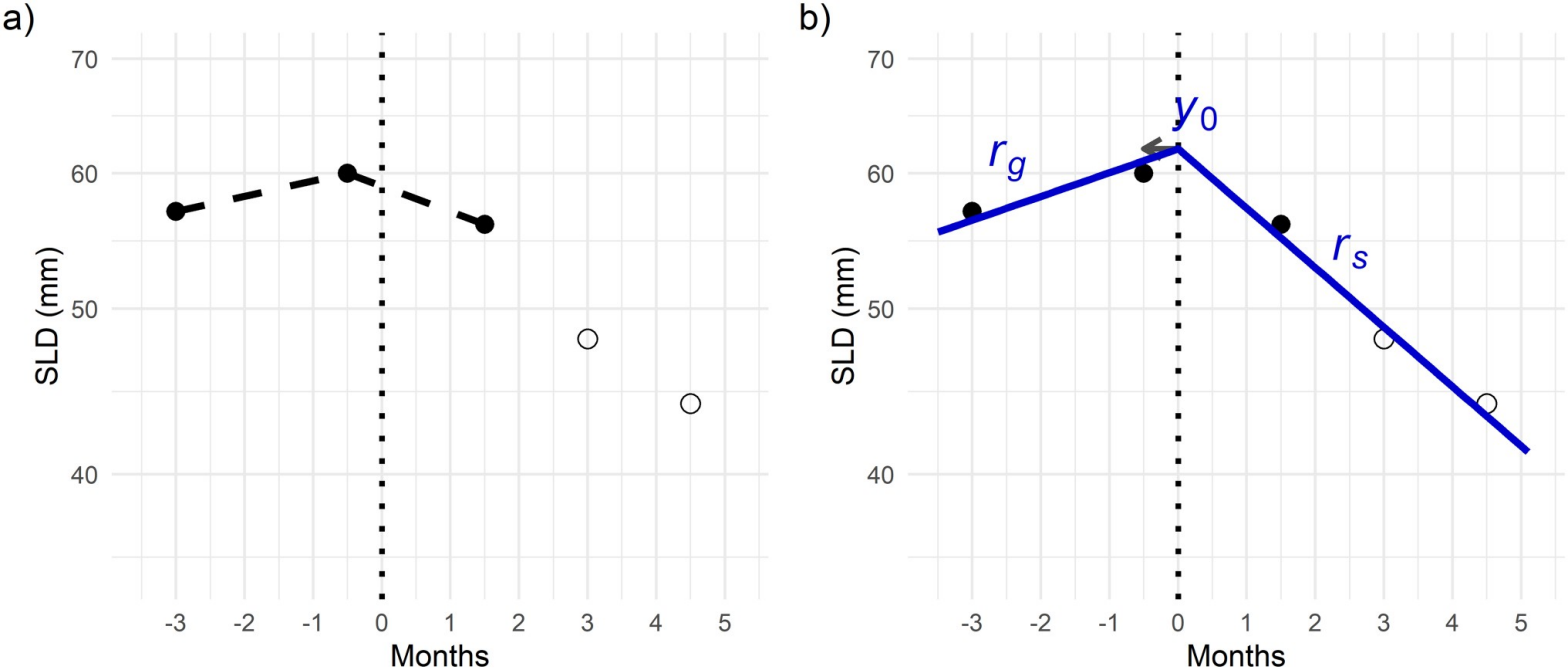
Model estimated vs. observed changes in SLD over time. [Created using R/ggplot2 [11]]. In these figures, the dots represent the observed SLD values (Fig 1a) and the blue curve depicts the model predicted time profile (segmented exponential function) (Fig 1b). If we had used the Ferté and colleagues approach (Fig 1a), the observations represented by open circles would have been omitted and the baseline would have been misplaced (as illustrated by the arrow).

A schematic illustrates the differences between the approach taken by Ferté and colleagues [4,5] (Fig 1a) and the model-based approach (Fig 1b) considered in the present analysis. The main differences are that, in the latter, (i) treatment effect was accounted for from treatment start (not before), (ii) all post-baseline scans contributed to the estimation of the on-treatment rate (rather than just the first). In addition, a selection of actual patients’ profiles is provided in S1 Fig, to further illustrate the differences between the approach supported by Ferté and colleagues and ours.

Negative values of *r*_*g*_ or *r*_*s*_ would represent tumor shrinkage, and positive values, tumor growth. A rate *r* could also be re-expressed and interpreted in terms of doubling or halving time for the tumor size using the equations *T*_*double*_ = ln(2)/*r* and *T*_*half*_ = ln(0.5)/*r*, respectively.

By looking at the absolute difference between the slope estimates in the treatment vs. reference period, i.e. *d* = *r*_*s*_ − *r*_*g*_, the drug effect could be assessed. In order to account for cases where the tumor dynamics of a given patient would not sensibly differ before and after treatment initiation values of *d* ranging between -0.05 (month^-1^) and +0.05 (month^-1^) were considered negligible. Such a cut-off of 0.05 is arbitrary and could be interpreted as a roughly 5% difference, per time increment, with the tumor size that would have been observed in absence of a drug effect.

## Results

Patient characteristics at baseline are summarized in Table 1. Of the 162 patients enrolled in the monotherapy arm, 156 had a measurable disease at baseline. Of those, 61 were PVNS patients and 95 non-PVNS. Among these 156 patients, 68 were qualified as *evaluable* for the analysis, as they had two pre-baseline scans and at least one TA during the treatment period.

**Table 1:** Patients characteristic at baseline.

	PVNS	Non-PVNS	Total
**All enrolled patients**			
N (row %)	61 (39.1)	95 (60.9)	156
Age (years)–median (range^b^)	38 (18–82)	62 (18–80)	53 (18–82)
Sex = Female–N (column %)	36 (59.0)	45 (47.4)	81 (51.9)
ECOG = 1 status–N (column %)^a^	8 (13.1)	67 (70.5)	75 (48.1)
**Patients with evaluable data**			
N (row %)	27 (39.7)	41 (60.3)	68
Age (years)–median (range^b^)	35 (18–82)	63 (30–80)	52 (18–82)
Sex = Female–N (column %)	15 (55.6)	22 (53.7)	37 (54.4)
ECOG = 1 status–N (column %)^a^	2 (7.4)	25 (61.0)	27 (39.7)

^a^ECOG = 1 (as opposed to ECOG = 0);

^b^range is (min-max)

The time window for collection of pre-baseline, baseline and post-baseline scans are presented in Table 2. The number of scans per patient ranged from a minimum of 3 to a maximum of 14. The median number of scans was higher in responders patients than in non-responders (Table 2).

**Table 2:** Summary statistics of information on timing and number of scans.

Endpoint	Median (range^b^)
Time of pre-baseline scan relative to treatment onset (months)	-2.60 (-12.0 to -0.86)
Baseline scan relative to treatment onset (months)	-0.40 (-1.19 to 0)
Number of post-baseline scans per evaluable patient	
- Responders^a^ (n = 16)	2 (1 to 3)
- Non-responders^a^ (n = 52)	1 (1 to 12)

^a^Responders are patients with best overall response (BOR) equal to PR or CR, else they are non-responders;

^b^range is (min to max)

**Fig 2:**
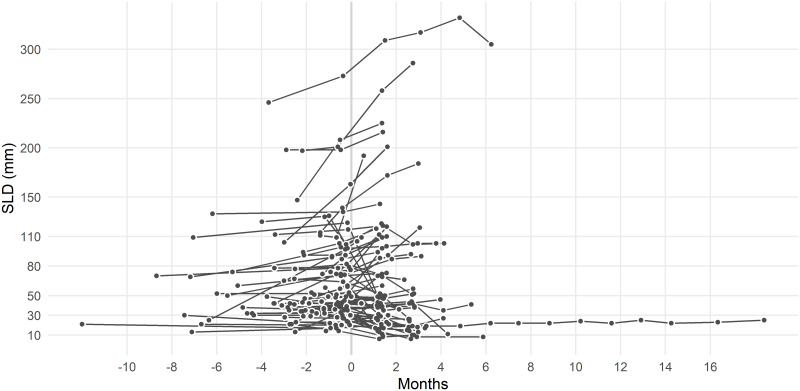
Individual time profiles for SLD values, N = 68. [Created using R/ggplot2 [11]].

The inter-individual variability in tumor size time profiles was large (Fig 2, see also the S2 Fig). The segmented regression line model adequately fit the data. The model goodness-of-fit can be judged based on the S3 Fig presenting the individual observations overlaid with the predicted curves for each evaluable subject.

The summary statistics of *r*_*g*_ and *r*_*s*_ are reported in Table 3. On average, the tumors appeared to be growing slowly (positive rate) in the reference (pre-treatment) period. The estimated median *r*_*g*_ was equal 0.022 month^-1^ (respectively 0.034 month^-1^ for the mean), and the pre-treatment median tumor size doubling time was estimated to 4.0 months. During the treatment period, the estimated median *r*_*s*_ was equal to 0.001 month^-1^ (respectively -0.062 month^-1^ for the mean). Despite the fact that the confidence interval of the mean difference in slopes (*d*) excluded 0 (thus suggesting a statistically significant effect), the magnitude of the average difference was still limited (mean of -0.096 month^-1^ and median of -0.060 month^-1^) (Table 3).

**Table 3:** Estimated monthly rates of change in tumor size before (*r*_*g*_) and after (*r*_*s*_) treatment onset, for all patients and by responder category.

Category	N	*r*_*g*_	*r*_*s*_	*d* = *r*_*s*_ − *r*_*g*_
		Mean [95% CI]	Mean [95% CI]	Mean [95% CI]
		Median	Median	Median
		(range^b^)	(range^b^)	(range^b^)
All patients	68	0.034 [-0.003, 0.071]	-0.062 [-0.139, 0.015]	-0.096 [-0.175, -0.017]
		0.022	0.001	-0.060
		(-0.468 to 0.517)	(-0.911 to 1.053)	(-0.933 to 1.077)
Responders^a^	16	-0.040 [-0.105, 0.025]	-0.454 [-0.565, -0.344]	-0.414 [-0.557, -0.272]
		-0.007	-0.426	-0.405
		(-0.427 to 0.133)	(-0.911 to -0.143)	(-0.933 to 0.167)
Non Responders^a^	52	0.057 [0.014, 0.100]	0.059 [-0.007, 0.124]	0.002 [-0.076, 0.079]
		0.038	0.047	-0.018
		(-0.468 to 0.517)	(-0.594 to 1.05)	(-0.750 to 1.08)

^a^Responders are patients with best overall response (BOR) equal to PR or CR, else they are non-responders.

^b^range is (min to max)

As expected, looking at the responder subgroup (per RECIST1.1), the difference between pre- and on-treatment phases was more pronounced with a mean *d* equal to -0.414 month^-1^ (95%CI ranging between -0.557 month^-1^ and -0.272 month^-1^) and a median equal to -0.405 month^-1^.

**Fig 3:**
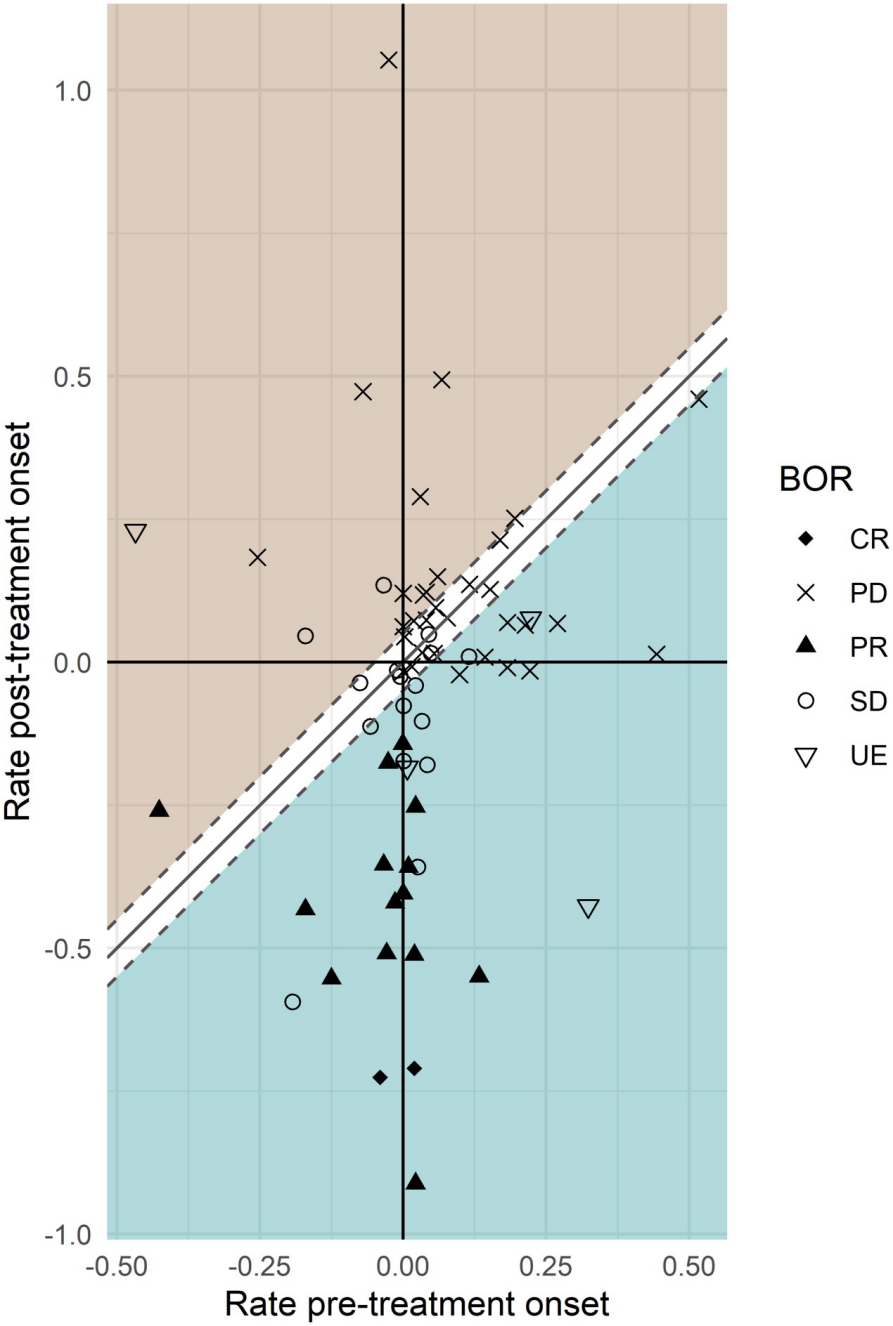
Individual rates of change in tumor size (in month^-1^) post- (*r*_*g*_) vs. pre- (*r*_*s*_) treatment onset, N = 68. [Created using R/ggplot2 [11]]. Points below the white area (*d* < −0.05 month^-1^) would represent an improvement after the beginning of therapy (i.e. tumor growth slows down, blue area) and in the opposite, points above the white area (*d* > 0.05 month^-1^) would represent a worsening of the condition, with the tumor growing faster after beginning of therapy (brown area). The white area (no coloring) in-between would represent cases where tumor growth is not perturbed by treatment. Best overall response (BOR) defined according to RECIST1.1 guideline as complete response (CR), partial response (PR), stable disease (SD), progressive disease (PD) or undefined (UE).

The change in slopes (*r*_*s*_ vs. *r*_*g*_) was also displayed in Fig 3 where we noticed that (i) all but one (a PR patient in the lower left quadrant) responder patients were below the identity line, and (ii) most of the SD patients were located near the identity line. The PD patients were scattered around the identity line; those who were improving actually all presented with new lesions, hence were categorized as PD according to RECIST 1.1. The observed and model-predicted time profile for the unexpected PR patient in the lower left quadrant is discussed in the S4 Fig.

**Fig 4:**
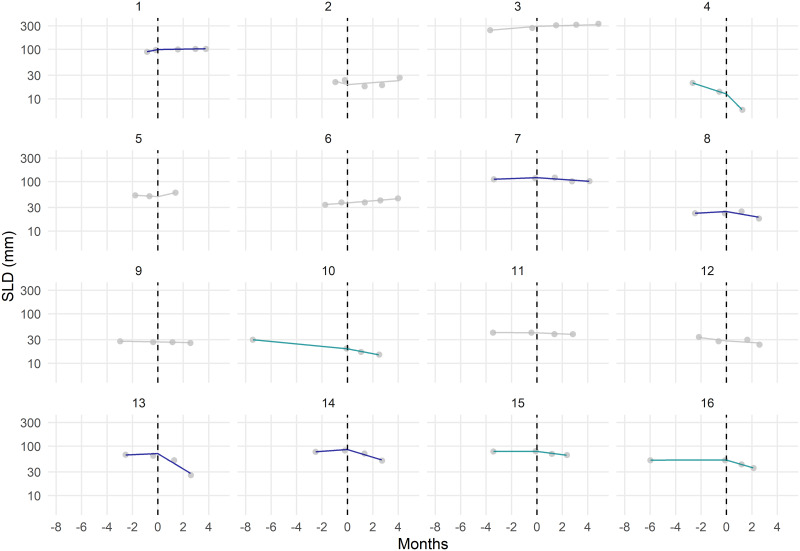
Observed and predicted tumor size in patients defined as SD according to RECIST1.1, N = 16. [Created using R/ggplot2 [11]]. Grey dots correspond to observed SLD values. Curves depict the model predicted longitudinal profiles. Patients who actually benefit from treatment (*d* < −0.05 month^-1^) are represented with either green or blue curves; patients who do not, are displayed with grey curves. Patients considered as having a *therapeutically induced stable disease* (*i*.*e*. *r*_*g*_ > 0.05 month^-1^ and *d* < −0.05 month^-1^) are in blue curves.

The correspondence between RECIST categories and difference in slopes is good overall (Table 4). All patients defined as PR or CR, but one, are improving. The PD category being dependent on the emergence of new lesion, no conclusion could be derived from the cross-distribution of patients in this BOR category. The SD category deserves more attention as differences in slopes actually bring some new insights. Among the sixteen SD patients, five have similar slopes before and after treatment, indicating that for those patients, the introduction of the new therapy did not change the tumor size dynamics. However, more than half of the SD patients (nine out of sixteen) demonstrate improvement with regard to the rate of change in tumor size (Fig 4). Defining anti-tumor response as ‘tumor growth declination’, the response rate would be 35.3% (24 out of 68), substantially higher than the response rate based on RECIST 1.1 (16/68 = 23.5%). Among the nine patients improving (as defined by *d* < −0.05 month^-1^), one had a positive and non-negligible pre-treatment tumor growth rate (*r*_*g*_ > 0.05 month^-1^). For this particular patient, the SD status was actually induced by the treatment.

**Table 4:** Correspondence between BOR CR, PR, or SD and categories (worsening, stable or improving) based on change in slopes from the tumor size model.

	Worsening n	Stable n	Improving n	Total N
CR^a^	0	0	2	2
PR^a^	1	0	13	14
SD^a^	2	5	9	16
PD^a^	11	11	9	31
Total	14	16	33	63^b^

^a^Best overall response (BOR) defined according to RECIST1.1 guideline as complete response (CR), partial response (PR), stable disease (SD), or progressive disease (PD).

^b^Five patients (out of 68) had undefined BOR category (UE).

## Discussion

The RECIST criteria are often used to assess antitumor activity of investigational agents in Phase 1 trials, but these criteria do not take into account the tumor growth rate prior to treatment start. Indeed, in this guideline, the anchor point to gauge any change in tumor size during a treatment period is the baseline SLD value. Anything happening before the baseline visit is disregarded. However, by ignoring the pre-baseline disease progression process, the pre-treatment tumor growth is implicitly considered as being null. In reality, an assessment of the rate of change in tumor size prior baseline can be made by having access to a MRI or CT scan collected at a date preceding the date of baseline visit. In this article, we report on an assessment of efficacy of a new therapeutic agent, emactuzumab, by considering the change in tumor size before versus after treatment onset, regardless of the tumor type, clinical measures other than tumor response, and mechanism of action of the investigational treatment. A segmented line regression model of longitudinal (logarithm-transformed) SLD values was used to retrospectively estimate the tumor growth prior to administration of an experimental therapy and its change thereafter. For each patient, the response to treatment was measured by the difference in slopes after versus before treatment onset.

At the population level, during the (pre-treatment) reference period, we observed a shallow median growth rate of 0.022 month^-1^ and a median tumor mass doubling time of approximately 4 months. This doubling time was shorter than the one reported by Talkington and Durrett (343 days, i.e. approximately 11 months) who evaluated the untreated growth of breast tumors in 23 women using data collected in 1979 [3]. It is worth mentioning that the inter-patient variability in growth rates was high, and if we were to focus on the patients with non-negative *r*_*g*_ values (44 patients), the median doubling time would be 13.5 months, i.e. closer to the 11 months observed by Talkington and Durrett. During the experimental period, we observed a trend opposite to the one observed in the reference period, with a negative exponential rate indicating tumor shrinkage on average. However, the rate of this tumor shrinkage was low (<0.05 month^-1^) and not considered as clinically meaningful overall. These low rates (during the pre-treatment or on-treatment periods) were actually masking large variations between patients, with rate estimates approximately ranging between -0.47 and +0.52 month^-1^ for *r*_*g*_ and -0.91 and +1.05 month^-1^ for *r*_*s*_.

We observed a good concordance between the proposed metric and RECIST category for PR and CR patients. For SD patients, the difference in tumor size time dynamics after vs. before treatment onset allowed us to re-classify 9 patients out of 16 from unresponsive (SD) to responsive. In those patients, the dynamics of tumor growth was not only stabilized but reversed. More specifically, a closer look at the individual tumor growth rates, allowed us to identify one patient (among the 9 responsive ones), whose tumor was growing pre-treatment, and stabilized during the treatment period, hence qualifying as therapeutically induced stable disease. The ability to distinguish therapeutically induced stable disease from cases where the tumor growth was unperturbed by the treatment may be one of the key benefits of assessing the post- versus pre-treatment rate for change in tumor dynamics evaluation.

Our study has a number of limitations: (1) the retrospective nature of our analyses warrants further validation, (2) the small size of the clinical trial limits the extension of our conclusions to the broader population, and (3) the data were obtained from a heterogeneous population of patients with different phenotypes and diagnoses. Another constraint with the proposed approach resides in the number of tumor assessments necessary to fit the model. Indeed, more than two pre-baseline and one post-baseline tumor assessment per patient would be needed to avoid a saturated model. In patients who discontinue the treatment after the first post-baseline tumor assessment (due to lack of efficacy), the model would just connect the dots and be useful for interpolation. In that sense, it would not add any value compared to an empirical slope calculation, based on the difference in observed SLD values divided by the elapsed time between tumor assessments.

Despite these limitations, our study supports the idea of extracting and using SLD values from pre-baseline scans to inform on the disease progression pattern of cancer patients in a clinical development setting. In our experience, collecting pre-baseline scans is feasible for most patients and requires minor additional costs, mainly imputable to the retrieval and analysis of the pre-treatment imaging. Measuring clinical benefit using the difference between exponential rates estimated in the experimental (on-treatment) versus reference (pre-treatment) period provides an additional value to standard RECIST measurements when determining the efficacy of targeted therapeutics in early-phase clinical studies as it (i) corrects for the natural growth rate of the tumor, and (ii) enables identification of patients who benefited from treatment within the SD cohort. Our findings warrant further exploration and validation of this approach as it could greatly facilitate early detection of drug efficacy and thereby support drug development.

## Supporting information

S1 Text (DOCX)

S1 Fig Illustration of the differences in the assessment of tumor dynamics using (i) the Ferté et al. approach, (ii) the model-based approach, for a selection of 11 patients. (DOCX)

S2 Fig Individual time profiles of SLD values for the 68 patients included in the analysis, according to their RECIST-based “responder” (n = 16) or “non-responder” status (n = 52). (DOCX)

S3 Fig Model-based individual time-SLD profile and the observations for all patients included in our analysis, N = 68. (DOCX)

S4 Fig Individual SLD values for the PR patient misclassified as ‘worsening’ by the proposed model (ID: 4073). (DOCX)
