# Supplementary material for: Therapeutically-induced stable disease in oncology early clinical trials
Source: PLoS One. 2020 May 29;15(5):e0233882. doi: 10.1371/journal.pone.0233882 (PMC7259628; doi:10.1371/journal.pone.0233882)
Supplement: S1 Text — (DOCX) [file pone.0233882.s001.docx]

**S1 Text**

Statistical analysis was performed in R (version 3.1.0). Using the *lmList* function of the “nlme” R package [1], a segmented line regression model was adjusted to the data of each patient $i$, in order to describe the changes in SLD values over time. The method used to estimate the model parameter was the ordinary least squares.

The regression model accepts two slopes for each patient $i$ respectively: one describing the change in SLD values over time before treatment onset ($r_{gi}$) and one after treatment onset ($r_{si}$). The treatment onset was used to set the time to 0, and served as fixed breakpoint in the segmented regression model; hence the difference in slopes was expressing the treatment effect. The model is expressed as follow:

$$y_{ij}^{'}=ln\left( y_{ij} \right)=ln\left( y_{0i} \right)+r_{gi}t_{ij}\cdot I\left( t_{ij}\leq0 \right)+r_{si}t_{ij}\cdot I\left( t_{ij}>0 \right)+\varepsilon_{ij}$$

with $y_{ij}$, the patient SLD (in mm) observed at the $j$^th^ occasion in patient $i$, $t$, the time in month, and $y_{0i}$, the intercept, *i.e*. the estimated SLD value for patient $i$ at time 0. In this equation, $I\left( \cdot\right)$ is the indicator function equal to 1 when the statement is true, and $\varepsilon_{ij}$ is the residual error expressing the deviation of each observation from the model.

The residual error terms were assumed to be normally distributed with mean 0 and pooled standard deviation $\sigma_{res}$. This pooled estimated residual standard deviation was obtained by adding together the residual sum of squares for each non-null element, dividing by the sum of the corresponding residual degrees-of-freedom, and taking the square root.
