# Supplementary material for: Therapeutically-induced stable disease in oncology early clinical trials
Source: PLoS One. 2020 May 29;15(5):e0233882. doi: 10.1371/journal.pone.0233882 (PMC7259628; doi:10.1371/journal.pone.0233882)
Supplement: S1 Fig — (DOCX) [file pone.0233882.s002.docx]

**S1 Fig. Illustration of the differences in the assessment of tumor dynamics using (i) the Ferté et al. approach, (ii) the model-based approach, for a selection of 11 patients.**

**
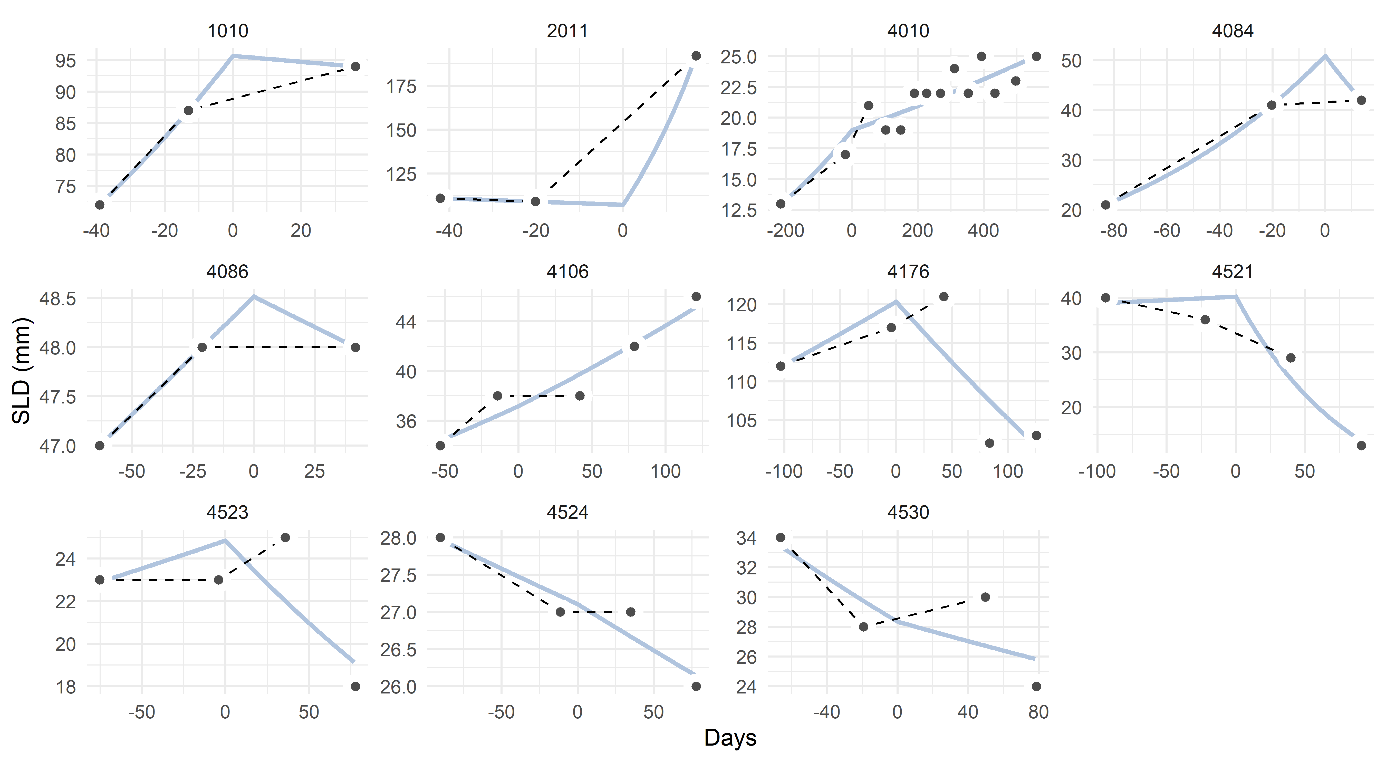
**

This plot illustrates the risk of slope miscalculation using Ferté and colleagues approach (dotted line) as opposed to the model-based approach (blue plain curves).
