# Supplementary material for: Therapeutically-induced stable disease in oncology early clinical trials
Source: PLoS One. 2020 May 29;15(5):e0233882. doi: 10.1371/journal.pone.0233882 (PMC7259628; doi:10.1371/journal.pone.0233882)

**S2 Fig. Individual time profiles of SLD values for the 68 patients included in the analysis, according to their RECIST-based “responder” (n=16) or “non-responder” status (n=52).**


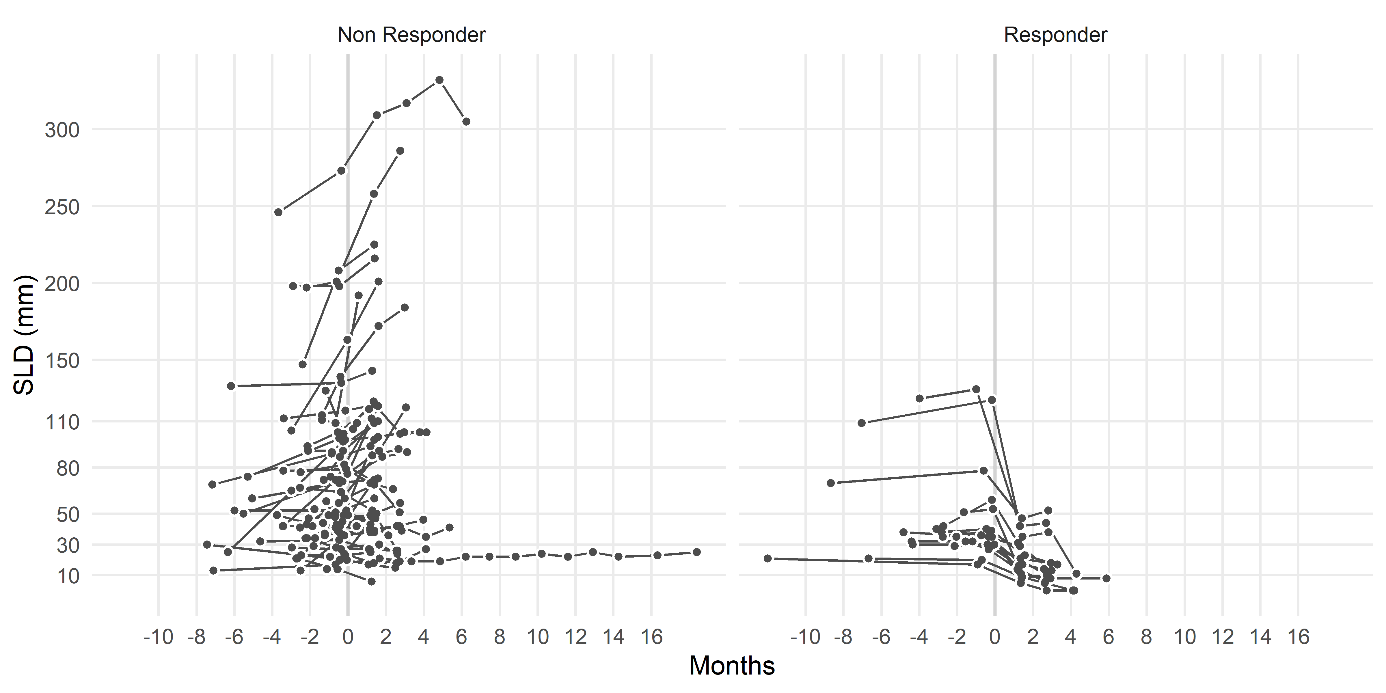

Supplement: S2 Fig — (DOCX) [file pone.0233882.s003.docx]
