# Supplementary material for: Therapeutically-induced stable disease in oncology early clinical trials
Source: PLoS One. 2020 May 29;15(5):e0233882. doi: 10.1371/journal.pone.0233882 (PMC7259628; doi:10.1371/journal.pone.0233882)
Supplement: S3 Fig — (DOCX) [file pone.0233882.s004.docx]

**S3 Fig. Model-based individual time-SLD profile and the observations for all patients included in our analysis, N=68.**

1/6


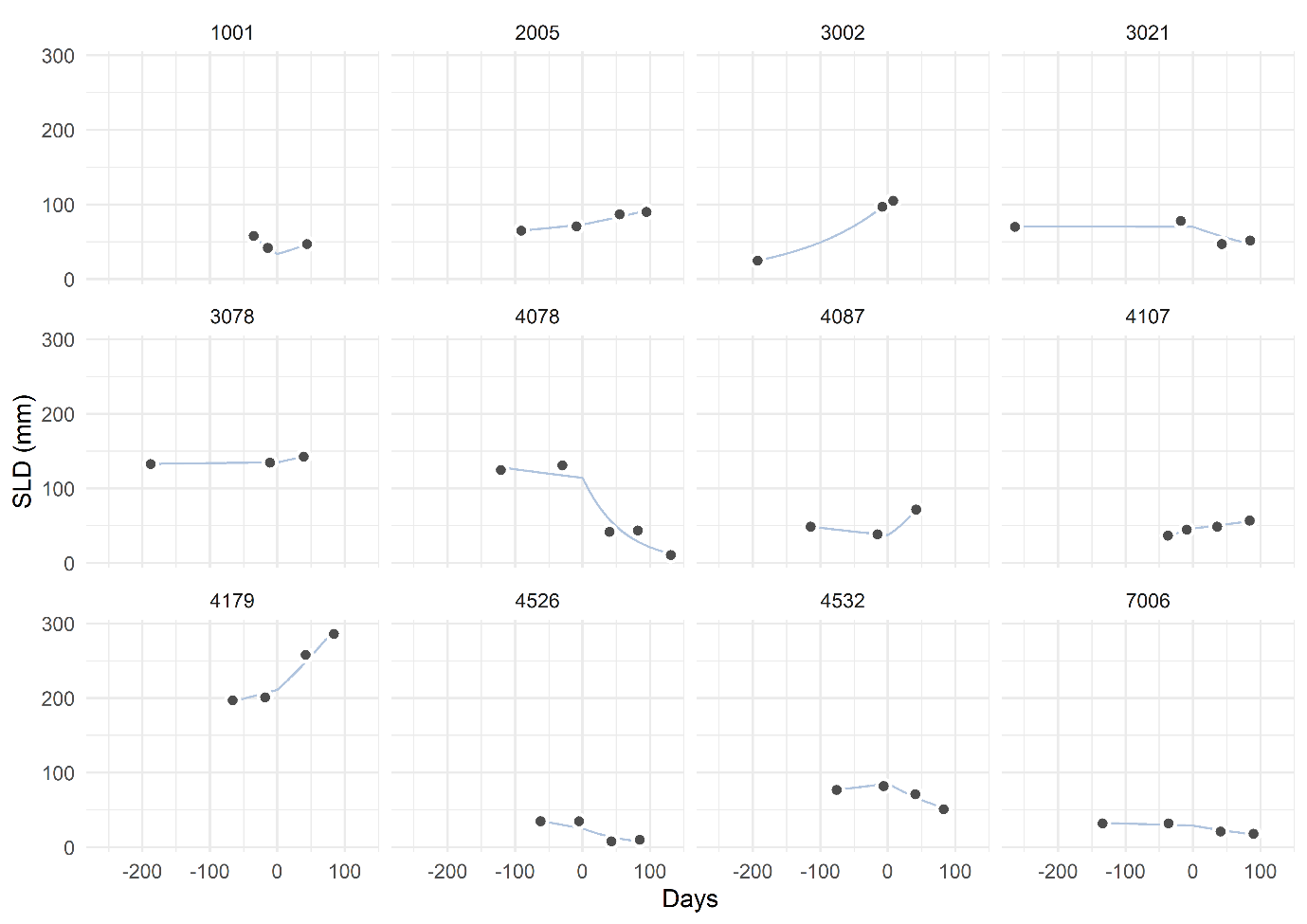


Dots represent the observed SLD values, line represent the predicted time trend for this patient.

2/6


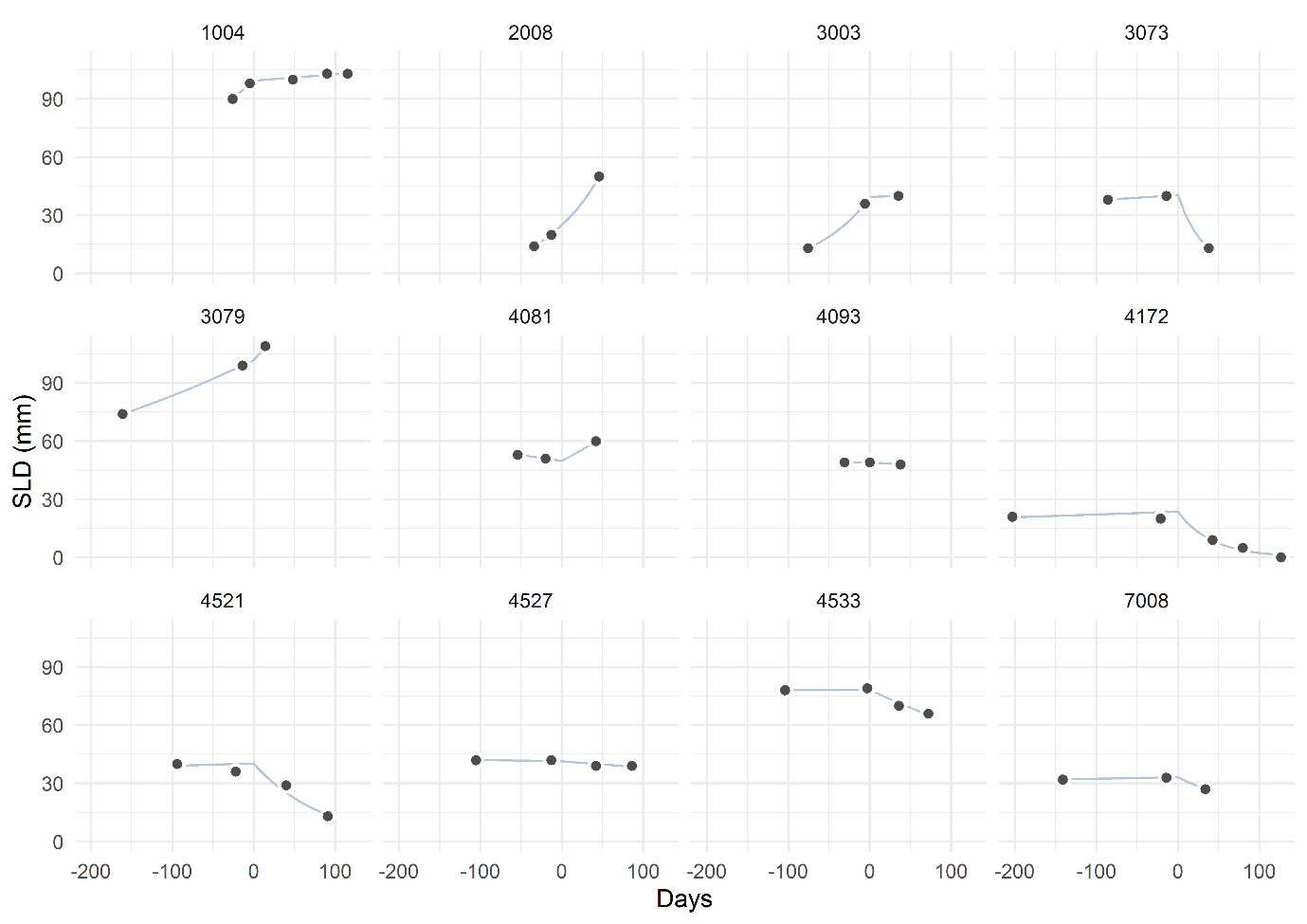


Dots represent the observed SLD values, line represent the predicted time trend for this patient.

3/6


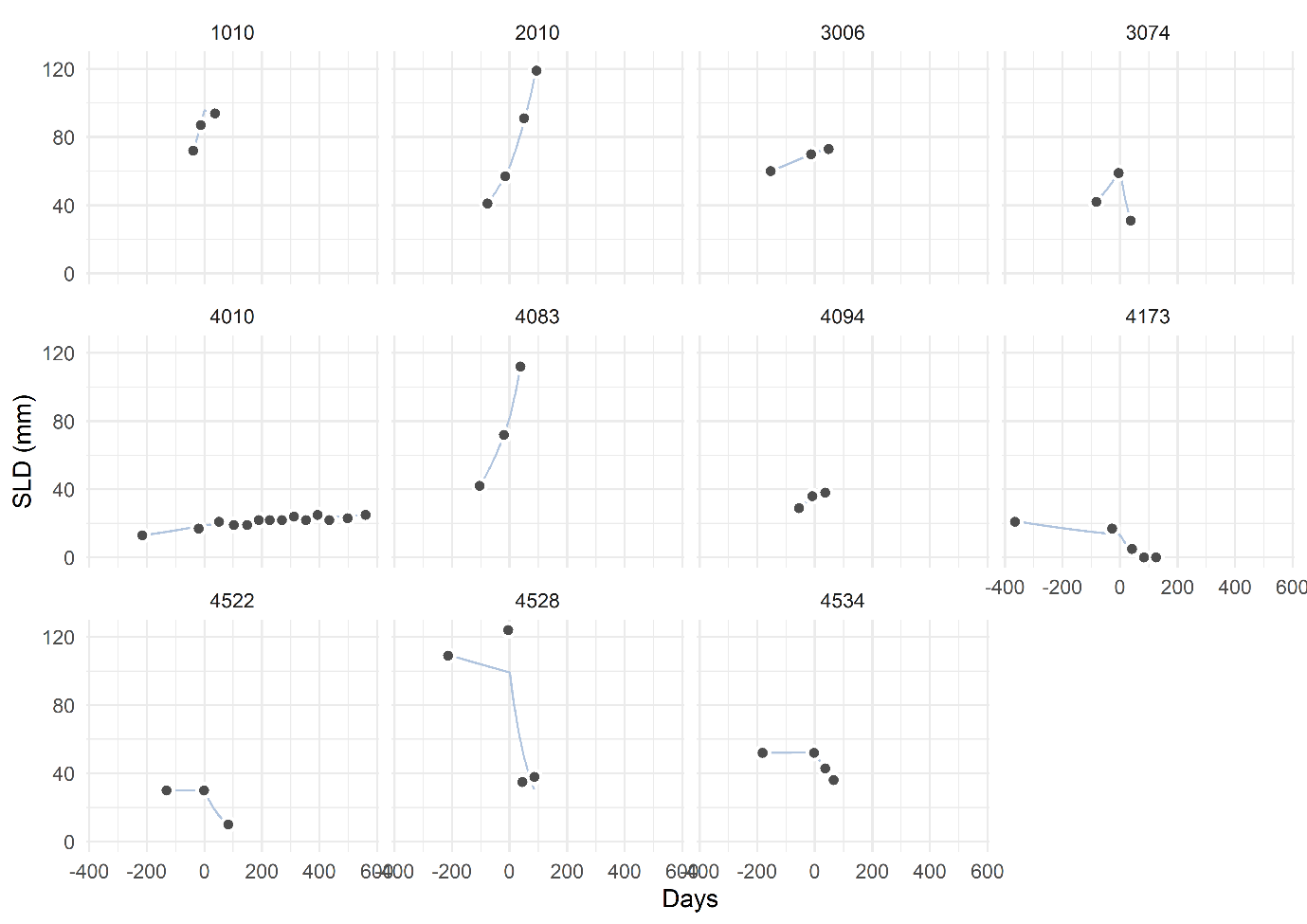


Dots represent the observed SLD values, line represent the predicted time trend for this patient.

4/6


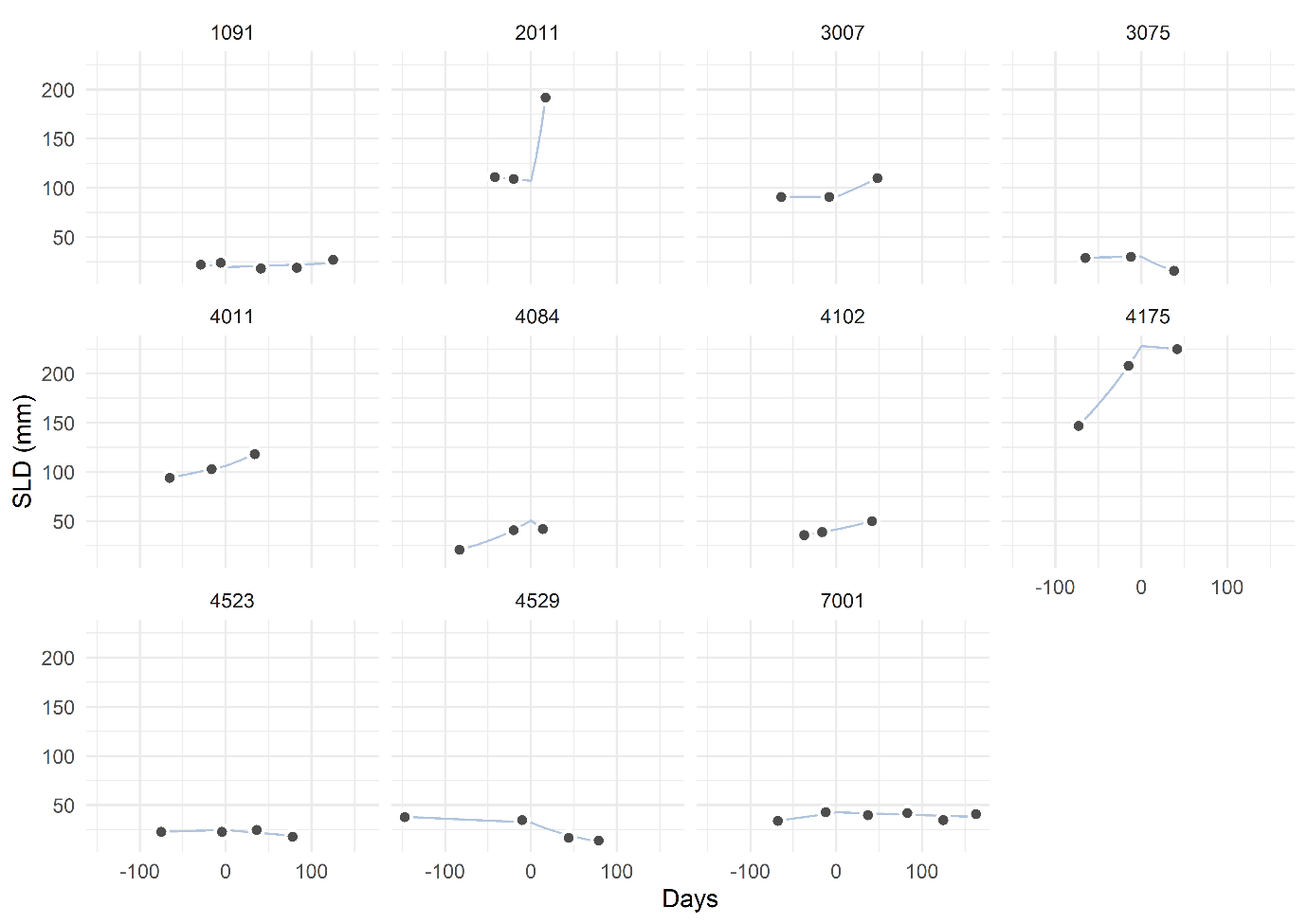


Dots represent the observed SLD values, line represent the predicted time trend for this patient.

5/6


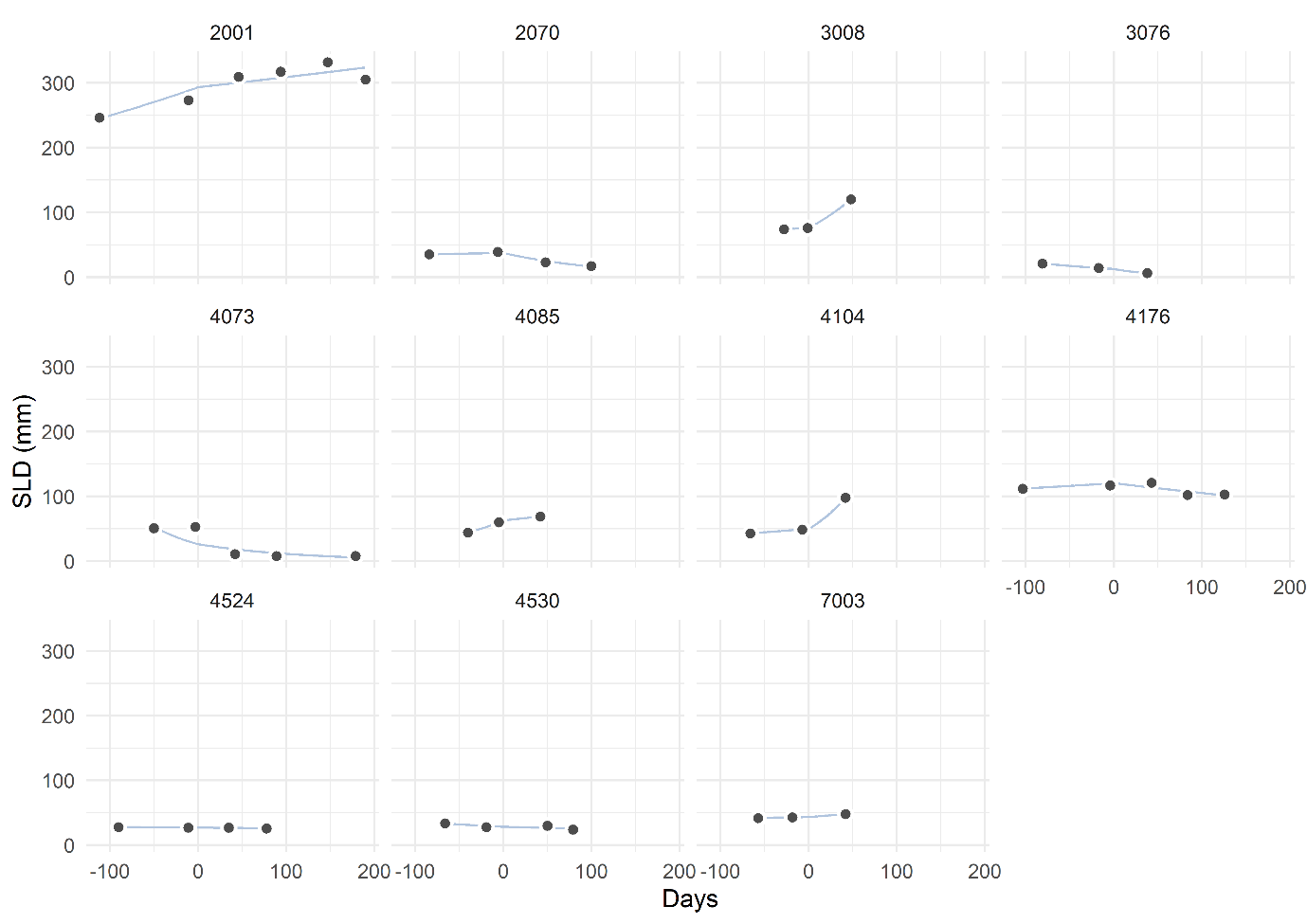


Dots represent the observed SLD values, line represent the predicted time trend for this patient.

6/6


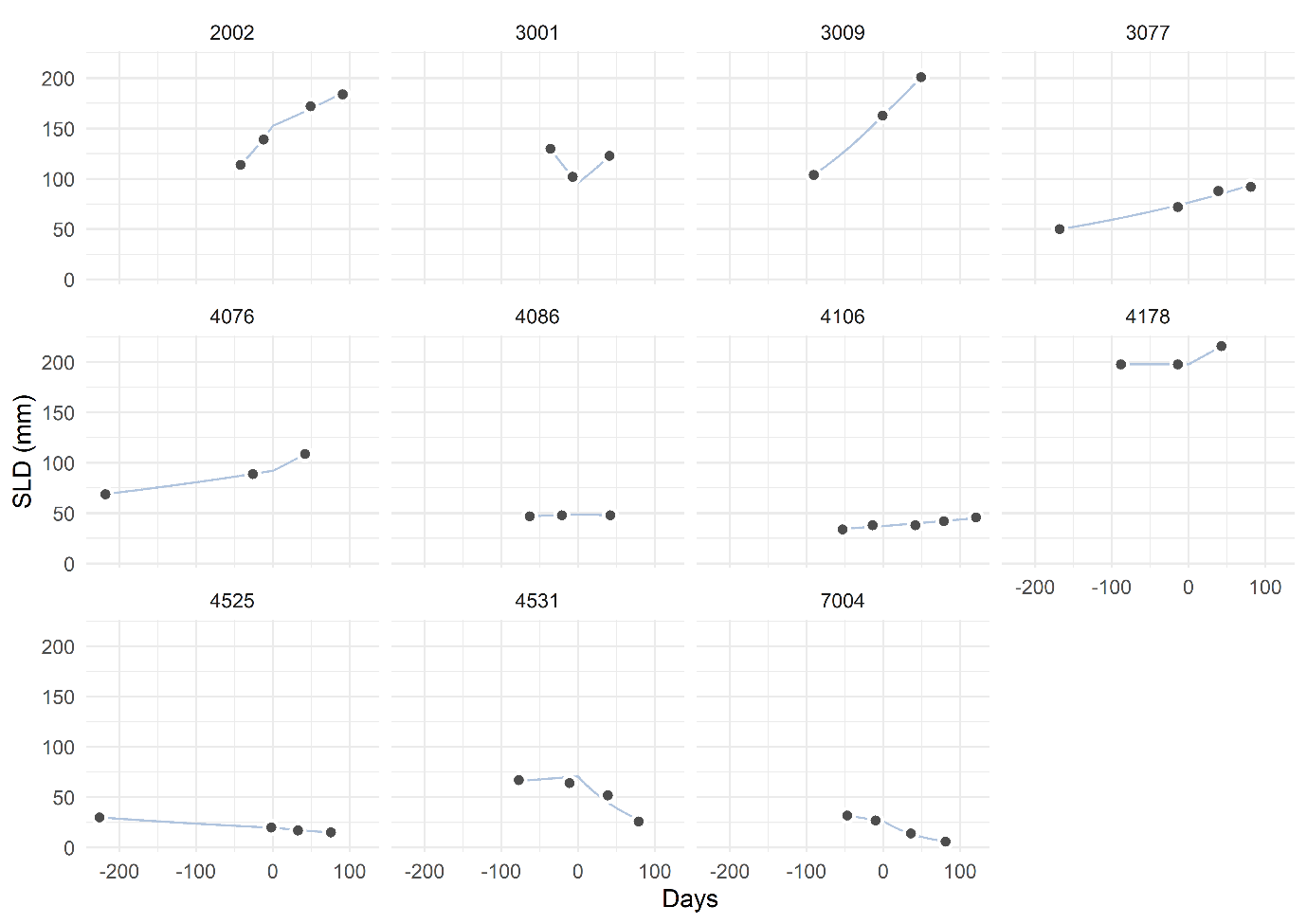


Dots represent the observed SLD values, line represent the predicted time trend for this patient.
