# Supplementary material for: Therapeutically-induced stable disease in oncology early clinical trials
Source: PLoS One. 2020 May 29;15(5):e0233882. doi: 10.1371/journal.pone.0233882 (PMC7259628; doi:10.1371/journal.pone.0233882)
Supplement: S4 Fig — (DOCX) [file pone.0233882.s005.docx]

**S4 Fig. Individual SLD values for the PR patient misclassified as ‘worsening’ by the proposed model (ID: 4073).**


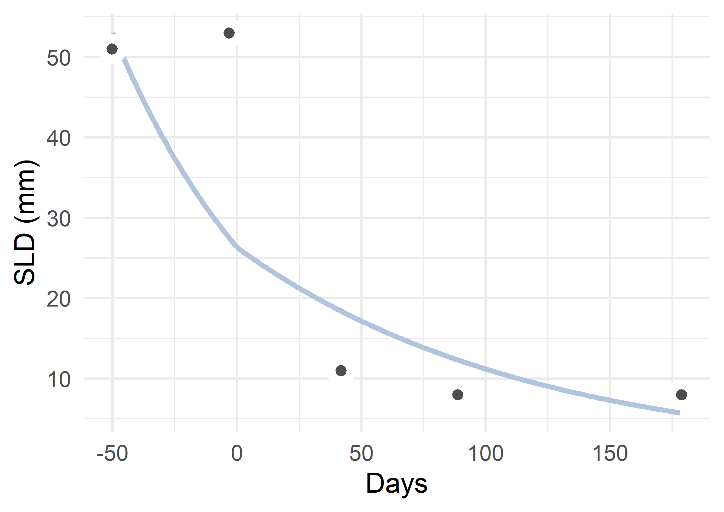


Dots represent the observed SLD values, line represent the predicted time trend for this patient.

This graph illustrates the case of a misclassification by the model. This is the only case observed in our analysis. The model is not able to fit the data closely due to the atypical trend in this patient, who has an immediate and sustained response to the treatment. The estimated $d$ for this patient is +0.17 mm^-1^, while it should have been negative.

While alternative models that are more flexible could have been considered to capture this type of response pattern (see reviews [2,3]), they are also more data demanding as they have more parameters. Hence, there is not optimal solution to this problem, except to report such misclassification cases.
